# Machine learning algorithms for identifying contralateral central lymph node metastasis in unilateral cN0 papillary thyroid cancer

**DOI:** 10.3389/fendo.2024.1385324

**Published:** 2024-05-10

**Authors:** Anwen Ren, Jiaqing Zhu, Zhenghao Wu, Jie Ming, Shengnan Ruan, Ming Xu, Tao Huang

**Affiliations:** ^1^ Department of Breast and Thyroid Surgery, Union Hospital, Tongji Medical College, Huazhong University of Science and Technology, Wuhan, China; ^2^ First Clinical College, Tongji Medical College, Huazhong University of Science and Technology, Wuhan, China

**Keywords:** papillary thyroid carcinoma, contralateral central lymph node metastasis, risk factors, prediction model, machine learning

## Abstract

**Purpose:**

The incidence of thyroid cancer is growing fast and surgery is the most significant treatment of it. For patients with unilateral cN0 papillary thyroid cancer whether to dissect contralateral central lymph node is still under debating. Here, we aim to provide a machine learning based prediction model of contralateral central lymph node metastasis using demographic and clinical data.

**Methods:**

2225 patients with unilateral cN0 papillary thyroid cancer from Wuhan Union Hospital were retrospectively studied. Clinical and pathological features were compared between patients with contralateral central lymph node metastasis and without. Six machine learning models were constructed based on these patients and compared using accuracy, sensitivity, specificity, area under the receiver operating characteristic and decision curve analysis. The selected models were then verified using data from Differentiated Thyroid Cancer in China study. All statistical analysis and model construction were performed by R software.

**Results:**

Male, maximum diameter larger than 1cm, multifocality, ipsilateral central lymph node metastasis and younger than 50 years were independent risk factors of contralateral central lymph node metastasis. Random forest model performed better than others, and were verified in external validation cohort. A web calculator was constructed.

**Conclusions:**

Gender, maximum diameter, multifocality, ipsilateral central lymph node metastasis and age should be considered for contralateral central lymph node dissection. The web calculator based on random forest model may be helpful in clinical decision.

## 1 Introduction

Thyroid cancer has now ranked as the 7th most common cancer globally, with an age-standardized incidence rates of 9.1 per 100 000 people in 2022 (1). Papillary thyroid cancer (PTC) constitutes the majority of thyroid cancer cases and significantly contributes to its growth (2). Surgery, including thyroidectomy and cervical lymph nodes dissection, TSH suppression therapy and radioiodine therapy are the core treatments of PTC. After comprehensive treatment, the majority of patients experience an ideal prognosis, while there are still more than 10% patients suffering relapse (3, 4).

Lymph node metastasis has been regarded as a prognostic factor in PTC, serving as a predictor for higher mortality and recurrence rates (5–7). Currently, the extent of lymph node dissection is mainly decided by the tumor size and clinical lymph node metastasis. However, some lymph nodes metastases are undetected using existing methods, especially the central lymph node metastasis (CLNM) due to their deep location and small size (8). It is reported that the rate of occult lymph node metastasis is around 24% to 82% (9). Occult lymph node metastasis may progress and lead to completion thyroidectomy, potentially impacting the quality of life of patients.

The incidence of contralateral cervical lymph node metastasis (CCLNM) in clinically negative lymph nodes (cN0) PTC patients is reported to range from 3.9% to 30.6%. Male gender, age < 45 years, lymphovascular invasion, extrathyroidal invasion, ipsilateral CLNM, multifocality, tumor size and tumor location are predictors for CCLNM (10–12). For cN0 patients, the necessity and extent of prophylactic central lymph node dissection (pCLND) are subjects of ongoing debate. Selective pCLND (13) and full extent pCLND (14) were recommended by different studies. Besides, other researchers suggested that ipsilateral CLND should be routinely performed, the decision to perform contralateral CLND should be based on intraoperative frozen-section pathology (15, 16), given the higher prevalence of complications such as permanent hypoparathyroidism in patients receiving bilateral CLND compared to those undergoing only ipsilateral CLND (17–21). Also, there are studies attempted to identify high-risk patients and proposed a “tailored” treatment (18), which means only performing pCLND in high-risk patients but not in others. However, not all factors have the same weight in predicting CCLNM, and a simple “yes or no” judgement is insufficient for clinical decision. Consequently, we attempted to constructed a more precise model to predict CCLNM.

Machine learning (ML), a rapidly evolving field in big data analysis, offers a more sophisticated approach to establish associations between input data and outcomes based on various data types, thereby providing more accurate predictions compared to traditional methods (22). Shortly after its appearance, researchers have been exploring the potential of machine learning to revolutionize medicine (23). Up to now, its application in medical research and practice is extensive. Many studies have explored using classification task to assist in diagnosis and predict prognosis (24, 25).

Here, we developed a machine learning model to predict CCLNM in unilateral cN0 PTC patients, evaluated its performance, and created an online calculator for easy assessment of CCLNM probability.

## 2 Materials and methods

### 2.1 Population and data collection

We retrospectively retrieved data of PTC patients from the Union Hospital of Tongji Medical College of Huazhong University of Science and Technology (Wuhan Union Hospital, WHUH) from 2009 to 2020 for training cohort and internal validation cohort. The external validation cohort was from the Differentiated Thyroid Cancer in China (DTCC) study [registered at ClinicalTrials.gov (NCT02638077)], including thyroid cancer patients from nine hospitals in China from 2014 to 2016 including The First Hospital of China Medical University, China-Japan Union Hospital of Jilin University, Chinese PLA General Hospital, First Affiliated Hospital of Kunming Medical University, West China Hospital, Tumor Hospital of Gansu Province, Sir Run Shaw Hospital, Wuhan Union Hospital and Tongji Hospital (26). The inclusion criteria were as follows: (1) no clinical evidence of lymph node metastasis; (2) total or near-total thyroidectomy with bilateral cervical lymph node dissection; (3) pathology proven unilateral PTC. The exclusion criteria were as follows: (1) distant metastasis before surgery; (2) incomplete clinical data; (3) history with other malignancy.

We retrospectively collected age, gender, body mass index (BMI), multifocality, maximum diameter, extrathyroidal evasion (ETE), and number of ipsilateral central lymph node dissection (No. of ICLND) and metastasis (No. of ICLNM) data of the enrolled patients. Age at diagnosis was divided into two groups: younger than 55 years and 55 years or older. According to WHO-BMI criteria, patients were divided into normal weight (18.0<= BMI <25.0), underweight (BMI <18.0), and overweight (BMI >=25.0). Maximum diameter was divided into <=1cm, >1 and <=4cm, and >4cm three groups. Ratio of ICLNM (RICLNM) was calculated as follows:


RICLNM(%)=No. of ICLNM/No. of ICLND

### 2.2 Development and comparison of ML-based models

Based on the presence or absence of CCLNM, patients were divided into two groups and their baseline information was compared. To further analyse the risk factors of CCLNM, we performed univariate and multivariate logistic regression analysis.

For construction and validation of ML models, we randomly split the data from WHUH into training cohort (80%) and validation cohort (20%). Six popular classification ML models were developed using all of the seven features, namely K-nearest neighbor (KNN), decision tree (DT), support vector machines (SVM), extreme gradient boosting (XGBoost), logistic regression (LR), and random forest (RF).

Multidimensional evaluation was used to evaluate the performance of the models, including accuracy, area under the receiver operating characteristic (AUC), sensitivity, specificity, false positive rate, and false negative rate. For accuracy, AUC, sensitivity and specificity, the closer to 1 they were, the better the model performed. While for false positive rate and false negative rate, the closer to 0 the better. To assess the clinical benefits of the models, decision curve analysis (DCA) was conducted. DCA is a method to demonstrate the net benefit, that is benefit to a true patient subtracts harm to a non-patient, of the treatment at certain threshold probability (27).

To interpret the models, we used feature importance to evaluate the contribution of variables to the models, and it was obtained by the increment of the prediction error of the model after rearranging according to the features.

### 2.3 Validation of the models

After choosing the best performed model, we validated it on internal validation cohort first and then external cohort. The assessment indicators were the same with those using for model comparisons. Confusion matrix was used to show the difference of the true situation and the predictive situation. Calibration curve was used to evaluate the agreement of truth and prediction.

### 2.4 Statistical analysis and web construction

All statistical analysis was performed by R software (The R Foundation for Statistical Computing). Chi-square test and Student’s t-test were used for categorical data and continuous data, respectively. Univariate and multivariate logistic regression analysis were performed to calculate the odds ratios (ORs) with 95% confidence intervals (*CI*s). *P* value <0.05 was considered to be statistically significant.

R package ‘shiny’ was used for a web calculator construction.

## 3 Results

### 3.1 Demographic and clinicopathological characteristics

**Figure 1:**
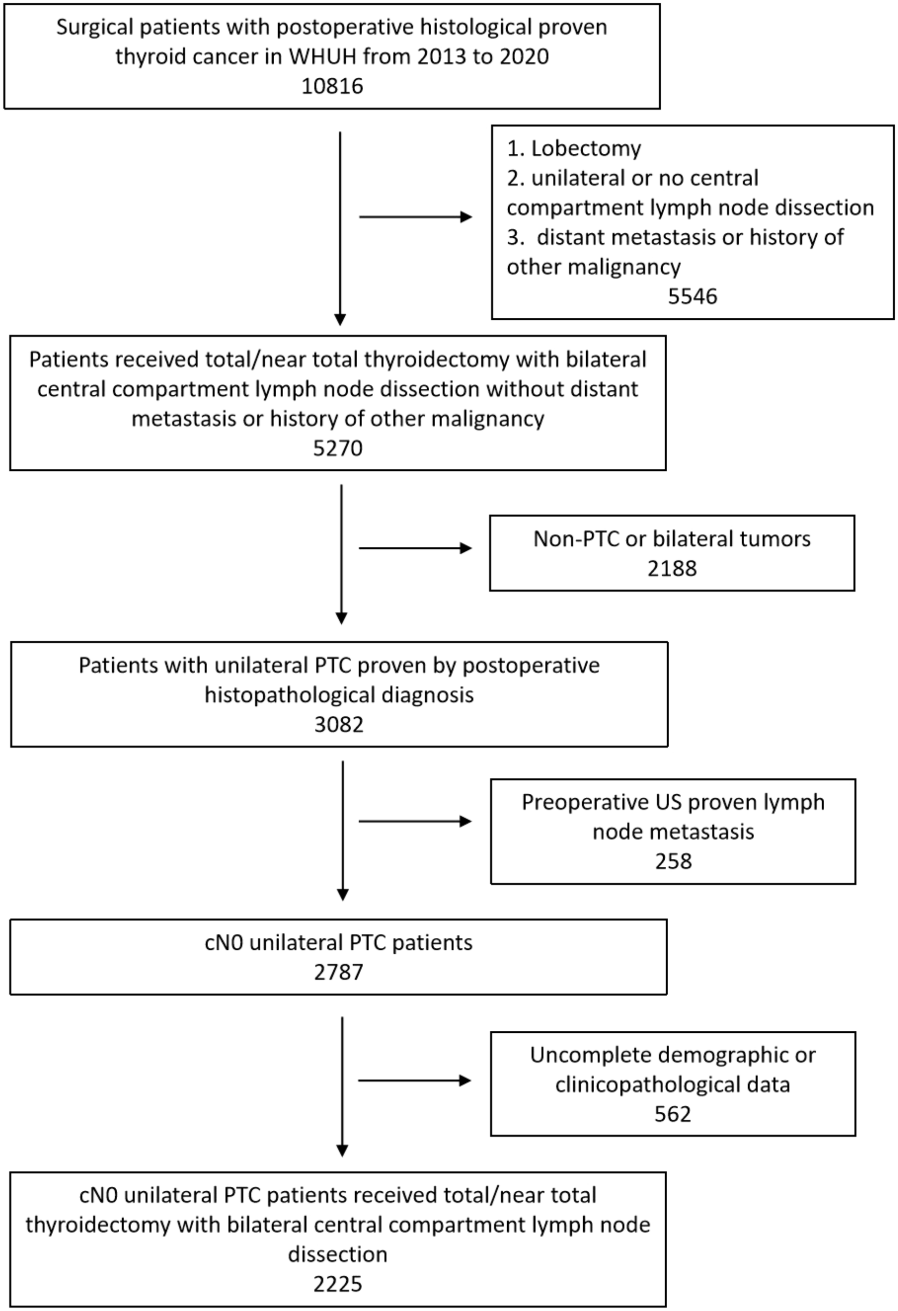
Flowchart of patient selection. PTC, Papillary thyroid carcinoma; US, Ultrasound.

There were 10816 patients underwent thyroid surgery from 2009 to 2020 in WHUH. A total of 2225 patients were included in the retrospective study (**Figure 1**). **Table 1** shows their demographic and clinicopathological characteristics. 1023 (46.0%) patients developed ICLNM while 469 (21.1%) patients developed CCLNM. Patients with CCLNM have demographic features like younger age (38.54 years V.S. 44.93 years, *P*<0.001) and male (33.3% V.S. 19.0%, *P*<0.001), while clinicopathological characteristics of CCLNM patients are larger tumor size (12.24mm V.S. 8.26mm, *P*<0.001), ETE (5.5% V.S. 3.1%, *P*=0.019), larger ratio of ICLNM (46.5% V.S. 13.7%, *P*<0.001). Multifocality and BMI did not show significant difference between the two group.

**Table 1:** Demographic and clinicopathologic features of the WHUH patients grouped by CCLNM.

	Levels	Total	Non-CCLNM ^a^	CCLNM	P value
		2225	1756	469	
Age Group (%)					<0.001
	<=55y	1896 (85.2)	1466 (83.5)	430 (91.7)	
	>55y	329 (14.8)	290 (16.5)	39 (8.3)	
Gender (%)					<0.001
	Female	1735(78.0)	1422 (81.0)	313 (66.7)	
	Male	490(22.0)	334 (19.0)	156 (33.3)	
BMI ^b^ Group (%)					0.386
	Normal Weight	1280 (57.5)	1018 (58.0)	262 (55.9)	
	Low Weight	112 (0.5)	83 (4.7)	29 (6.2)	
	High Weight	833 (37.4)	655 (37.3)	178 (38.0)	
Multifocality (%)					0.148
	No	1809 (81.3)	1439 (81.9)	370 (78.9)	
	Yes	416 (18.7)	317 (18.1)	99 (21.1)	
Maximum Diameter Group (%)					<0.001
	<=1cm	1498 (67.3)	1272 (72.4)	226 (48.2)	
	<=4cm	716 (32.2)	482 (27.4)	234 (49.9)	
	>4cm	11 (0.5)	2 (0.1)	9 (1.9)	
ETE ^c^ (%)					0.019
	No	2144 (96.4)	1701 (96.9)	443 (94.5)	
	Yes	81 (3.6)	55 (3.1)	26 (5.5)	
ICLNM ^d^ (%)					<0.001
	No	1202 (54.0)	1109 (63.2)	93 (19.8)	
	Yes	1023 (46.0)	647 (36.8)	376 (80.2)	
					
Age (years, mean (SD))		43.58 (11.20)	44.93 (10.82)	38.54 (11.20)	<0.001
BMI (mean (SD))		23.25 (3.37)	23.25 (3.39)	23.23 (3.30)	0.887
Maximum Diameter (mm, mean (SD))		9.10 (6.42)	8.26 (5.64)	12.24 (8.00)	<0.001
ICLND ^f^ (mean (SD))		7.23(4.38)	7.22 (4.40)	7.28 (4.29)	0.013
RICLNM ^e^ (mean (SD))		0.21 (0.29)	0.14 (0.23)	0.46 (0.33)	<0.001

^a^ CCLNM, contralateral center lymph node metastasis; ^b^ BMI, Body mass index; ^c^ ETE, extrathyroidal evasion; ^d^ ICLNM, ipsilateral central lymph node metastasis; ^e^ RICLNM, ratio of ICLNM; ^f^ ICLND, ipsilateral central lymph node dissected.

### 3.2 Feature selection

**Figure 2:**
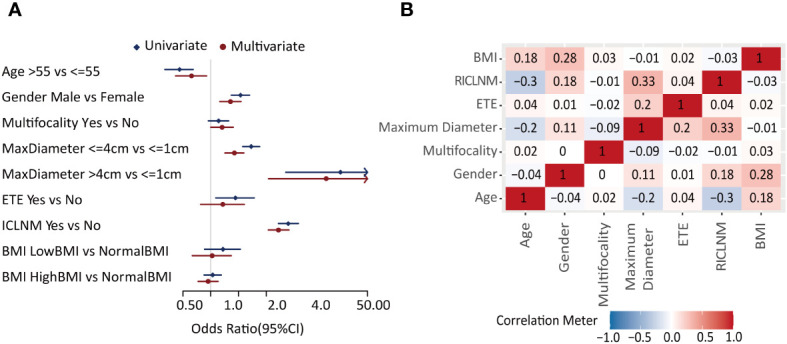
Feature selection. **(A)** Forest plot of the univariate and multivariate analysis of factors in predicting CCLNM. **(B)** Correlation analysis of each two factors. CCLNM, contralateral center lymph node metastasis; BMI, Body mass index; ETE, extrathyroidal evasion; ICLNM, ipsilateral central lymph node metastasis; RICLNM, ratio of ICLNM.

Univariate analysis showed age, gender, maximum diameter, ETE, and ICLNM were significantly related to CCLNM (*P*<0.05), whereas multifocality and BMI did not show obvious difference. In multivariate analysis, male (OR= 1.651, 95%*CI*: 1.267 -2.148, *P*<0.001), multifocality (OR= 1.325, 95%*CI*: 1.000 -1.747, *P*=0.048), maximum diameter between 1 to 4cm (OR= 1.799, 95%*CI*: 1.424 -2.274, *P*<0.001), maximum diameter larger than 4cm (OR= 17.747, 95%*CI*: 4.207 -122.140, *P*<0.001), and ICLNM (OR= 5.436, 95%*CI*: 4.216 -7.066, *P*<0.001) were independent risk factors of CCLNM. Whereas older age (OR=0.619, 95%*CI*:0.416-0.899, *P*=0.014) is a protective factor (**Figure 2A**, **Supplementary Table 1**).

Correlation analysis (**Figure 2B**) showed that maximum diameter and RICLNM have strong correlation (>0.03), while there is not significant correlation between any other two features. Considering their clinical significance, we included all factors above into ML models.

### 3.3 Performance of machine learning algorithms

**Figure 3:**
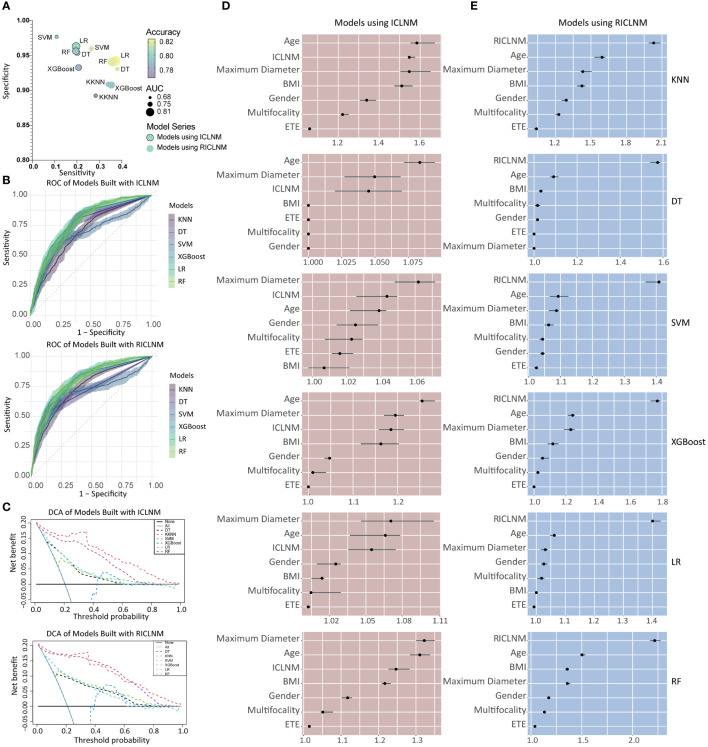
Comparison of different models built with ICLNM and RICLNM. **(A)** Bubble plot showing the sensitivity, specificity, accuracy and AUC of 12 models. **(B)** ROC of models built with ICLNM (upper) and RICLNM (lower). **(C)** DCA of models built with ICLNM (upper) and RICLNM (lower). **(D)** Feature importance of models built with ICLNM. **(E)** Feature importance of models built with RICLNM. BMI, Body mass index; ETE, extrathyroidal evasion; CCLNM, contralateral center lymph node metastasis; ICLNM, ipsilateral central lymph node metastasis; RICLNM, ratio of ICLNM; KNN, K-nearest neighbor; DT, decision tree; SVM, support vector machines; XGBoost, extreme gradient boosting; LR, logistic regression; RF, random forest.

Using age, gender, BMI, maximum diameter, multifocality, ETE, and ICLNM, predictive models for CCLNM were developed based on 6 algorithms, namely KNN, DT, SVM, XGBoost, LR, and RF. To compare the predictive value of ICLNM and RICLNM, we built another 6 models using age, gender, BMI, maximum diameter, multifocality, ETE, and RICLNM. **Supplementary Table 2** detailed the 12 models. Comparisons of their performance on the training cohort were demonstrated in **Figure 3A** and ROC in **Figure 3B**. All of the models had excellent accuracy, AUC and specificity, with LR having the highest accuracy (0.801) and AUC (0.786). When using RICLNM to build the models, better accuracy, AUC and sensitivity were achieved, with slightly lower specificity. DCA was performed to evaluate the clinical utility of these models (**Figure 3C**). RF and KNN showed obvious higher net benefits than others. For better understanding the models, relative importance of the features was shown in **Figures 3D**, **E**. It is interesting that RICLNM showed great importance in all 6 models built with it, while in models built with ICLNM it is not always the most important one.

Due to its great performance on accuracy, AUC and DCA, RF using RICLNM was chosen as a potential model for predicting CCLNM.

### 3.4 Predictive performance of RF on internal validation cohort

**Figure 4:**
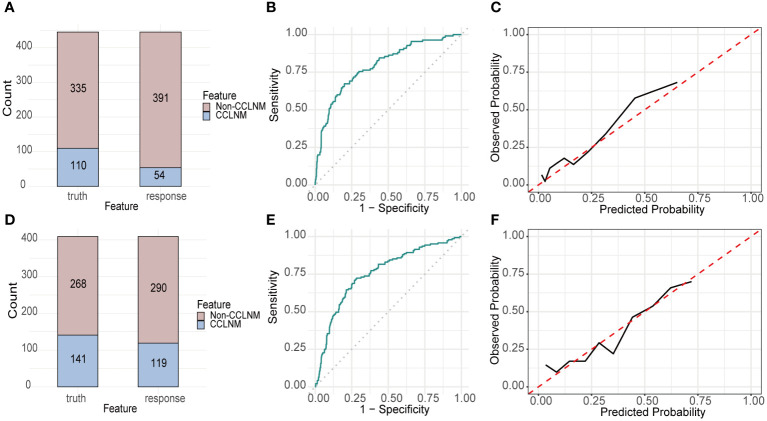
Performance of RF built with RICLNM on internal and external validation cohort. Confusion matrix **(A)**, ROC **(B)** and calibration curve **(C)** of internal validation cohort. Confusion matrix **(D)**, ROC **(E)** and calibration curve **(F)** of external validation cohort. CCLNM, contralateral center lymph node metastasis.

The validation cohort was used to prove the predictive performance of RF model and it is similar to the training cohort (accuracy 0.807, AUC 0.793, sensitivity 0.355, specificity 0.955). The confusion matrix and ROC were shown in **Figures 4A, B**, respectively. The calibration curve (**Figure 4C**) demonstrated good agreement between prediction and observation.

### 3.5 Predictive performance of RF on external validation cohort

**Figure 5:**
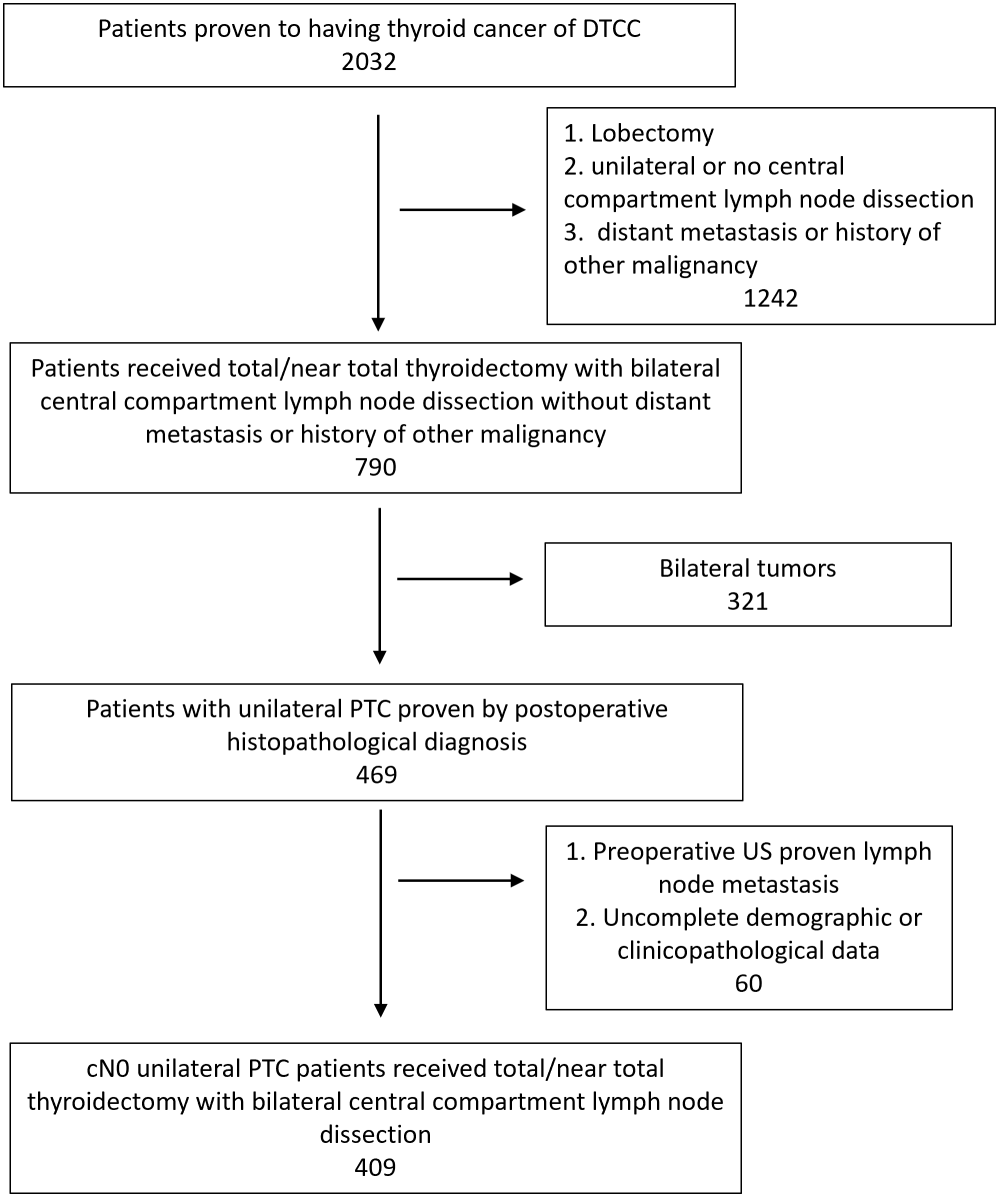
Flowchart of patient selection of external validation cohort. PTC, Papillary thyroid carcinoma; US, Ultrasound.

**Figure 5** showed the selection of external validation cohort. **Table 2** summarized the demographic and clinicopathological features of the 409 selected patients from DTCC cohort. The CCLNM rate is higher than data from our center (34.5% V.S. 21.1%) and patients of DTCC cohort have larger maximum diameter (15.21mm V.S. 9.10mm), higher ETE rate (21.52% V.S. 3.60%), and higher ratio of ICLNM rate (0.39 V.S. 0.21).

**Table 2:** Demographic and clinicopathologic features of the DTCC patients grouped by CCLNM.

	Levels	Total	Non-CCLNM ^a^	CCLNM	p
		409	268	141	
Age Group (%)					0.906
	<=55y	363 (88.8)	237 (88.4)	126 (89.4)	
	>55y	46 (11.2)	31 (11.6)	15 (10.6)	
Gender (%)					0.001
	Female	302 (73.8)	213 (79.5)	89 (63.1)	
	Male	107 (26.2)	55 (20.5)	52 (36.9)	
BMI ^b^ Group (%)					0.400
	Normal Weight	151 (36.9)	101 (37.7)	50 (35.5)	
	Low Weight	16 (4.9)	8 (3.0)	8 (5.7)	
	High Weight	242 (59.2)	159 (59.3)	83 (58.9)	
Multifocality (%)					1.000
	No	365 (89.2)	239 (89.2)	126 (89.4)	
	Yes	44 (10.8)	29 (10.8)	15 (10.6)	
Maximum Diameter Group (%)					0.002
	<=1cm	188 (46.0)	139 (51.9)	49 (34.8)	
	<=4cm	209 (51.1)	124 (46.3)	85 (60.3)	
	>4cm	12 (2.9)	5 (1.9)	7 (5.0)	
ETE ^c^ (%)					0.292
	No	221 (54.0)	215 (80.2)	106 (75.2)	
	Yes	88 (21.5)	53 (19.8)	35 (24.8)	
ICLNM ^d^ (%)					<0.001
	No	117 (28.6)	100 (37.3)	17 (12.1)	
	Yes	292 (71.4)	168 (62.7)	124 (87.9)	
					
Age (mean (SD))		41.82 (11.83)	43.15 (11.44)	39.29 (12.18)	0.002
BMI (mean (SD))		23.33 (3.32)	23.33 (3.31)	23.32 (3.36)	0.972
Maximum Diameter (mean (SD))		15.22 (10.91)	13.65 (9.81)	18.21 (12.23)	<0.001
RICLNM ^e^ (mean (SD))		0.39 (0.34)	0.29 (0.30)	0.58 (0.33)	<0.001

^a^ CCLNM, contralateral center lymph node metastasis; ^b^ BMI, Body mass index; ^c^ ETE, extrathyroidal evasion; ^d^ ICLNM, ipsilateral central lymph node metastasis; ^e^ RICLNM, ratio of ICLNM.

Accuracy, AUC, sensitivity and specificity of RF on DTCC cohort are 0.731, 0.755, 0.532 and 0.836, respectively. **Figures 4D–F** demonstrated the confusion matrix, ROC and calibration curve of RF on DTCC cohort.

### 3.6 Web calculator

**Figure 6:**
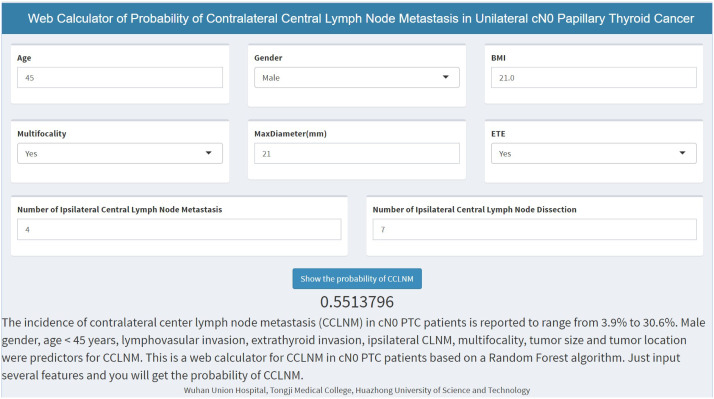
Web calculator based on the random forest model.

For conveniently calculating the CCLNM probability in clinical practice, we established an online calculator based on RF model (https://cclnm.shinyapps.io/CCLNMAPP/). Clinicians can predict the CCLNM risk by simply inputting 7 variables (**Figure 6**).

## 4 Discussion

In this study, we developed and compared 6 popular machine learning algorithms-based models to predict CCLNM in cN0 unilateral PTC patients using multicenter clinical data, utilizing demographic and clinicopathologic features. RF algorithm was selected for further validation and web calculator construction due to its outstanding performance in terms of accuracy, AUC and DCA. Both internal and external validation of the model were performed and showed satisfying results, indicating its potential for widespread application. A web calculator was constructed to facilitate the estimation of the probability of CCLNM in cN0 unilateral PTC patients.

This model helps in identifying CCLNM patients using demographic and clinicopathologic features which are easy to obtained before and during operation. Our study had large population (1780 patients in training cohort, 445 in internal validation cohort, and 409 in external validation cohort), which provides more precise prediction. Besides, to reduce selection bias, our study conducted the validation in external cohort using multicenter clinical data from nine different hospitals. With promising performance, our model showed great robustness and extensive application in CCLNM prediction. Most studies only identified some risk factors of CCLNM in cN0 PTC patients, which were not applicable enough in clinical decision. We not only analyzed the predictive value of several factors, but also constructed a prediction model, and the utility of machine learning makes the prediction more accurate.

The prevalence of CCLNM in our study was 21.1%, which was consistent with previous studies (8.13%-34.3%) (11, 28, 29). The discrepancy may be attributed to variations in patient populations and surgical criteria. We identified that <55 years, male, tumor size > 1cm, multifocality, and ICLNM were risk factors of CCLNM in cN0 PTC patients, while ETE showed no significant prediction value, in line with previous studies (10).

We investigated several demographic factors including age, gender and BMI in this work. It is widely accepted that older age is associated with poorer prognosis in thyroid cancer patients (30), but in our study and previous studies younger age were identified as a predictor of CCLNM (10). Furthermore, studies for total CLNM yielded similar results (31). Although PTC is more prevalent in women than men, male has been reported to be a risk factor of poorer prognosis and worse response to treatment. The underlying molecular mechanisms remains unclear but estrogen and androgen may play a role (32). Even though obesity is reported to increase thyroid cancer incidence (33), its role in lymph node metastasis is contradictory (34, 35) and differs depending on the regions (36). The relationship between obesity and lymph node metastasis and the mechanism underlying requires further study.

Some of the clinicopathologic features showed effects in CCLNM prediction in this work. Our results indicated that in cN0 unilateral PTC patients, multifocality was important to predict CCLNM. Multifocality relates to advanced disease and indicates to higher rate of recurrence, and thus patients with more than one lesions should receive more aggressive treatment (37). 2015 ATA guideline took tumor size into consideration when determining whether to perform pCLND (2). As many other studies (11, 29, 38–41), our results indicated the maximum tumor size was significantly larger in patients with CCLNM than those without. The relationship of ETE and thyroid cancer prognosis is controversial and recent studies further divided it into minimal ETE and extensive ETE based on the extent involved, which showed differences in clinicopathological features, like lymph node metastasis and prognosis (42, 43). As for CCLNM, previous study also demonstrated contradictory results (29, 38, 40). Our analysis revealed that ETE had significant value when performing univariate logistic regression, but not in multivariate analysis, indicating it was not an independent risk factor. However, we did not classify ETE into minimal and extensive group, which may lead to different outcomes.

Similar to previous studies (28, 29, 38–40), ICLNM exhibited great predictive value for CCLNM. Despite of the presence of “skip” metastasis, most metastasis occurs ipsilaterally first. It is interesting that when the models were developed using RICLNM instead of ICLNM, their sensitivity and performance in DCA increased dramatically, with only slight decrease of specificity, and in those models RICLNM had the strongest importance among all variables. Most previous study only focused on the existence of ICLNM, while only Zhou and Qin (11) included the amount of ICLNM in their analysis. Due to the different extent of dissection, absolute number of lymph nodes with metastasis may not reflect the true situation. Studies have demonstrated the significance of metastatic lymph node ratio (MLNR) in PTC prognosis (44–46) but it is rarely used in predicting CCLNM up to now. Our study showed that RICLNM was a stronger predictor of CCLNM than the existence of ICLNM. However, when only very few suspicious lymph nodes are dissected, the ratio will be either extremely large or small. The relationship between the extent of lymph node dissection and clinical value of MLNR requires further study.

There are some limitations of our study. First, its retrospective nature introduced unavoidable bias. Prospective studies are required to verify the accuracy and clinical benefit of the model. Second, although we externally validated of the model using DTCC cohort which includes data from nine centers, patients were all from China. Its application in other races needs further validation. Third, all histopathological features, including maximum diameter, multifocality, ETE and ICLNM, in the study were postoperative results, which are difficult to acquire preoperatively with current detection methods, but a rapid frozen pathological examination intraoperatively can offer the characteristics in need. Fourthly, since the clinical classification is operator-dependent, the judgement of cN0 is not absolutely objective and consistent. More accurate imaging methods may solve this problem in the future.

In conclusion, we presented a ML-based model to predict CCLNM probability in cN0 unilateral PTC patients, validated it in internal and external cohort, and developed an easy-to-use web calculator based on it.

## Data availability statement

The original contributions presented in the study are included in the article/**Supplementary Material**. Further inquiries can be directed to the corresponding authors.

## Ethics statement

Ethical approval was not required for the study involving humans in accordance with the local legislation and institutional requirements. Written informed consent to participate in this study was not required from the participants or the participants’ legal guardians/next of kin in accordance with the national legislation and the institutional requirements.

## Author contributions

AR: Conceptualization, Formal analysis, Methodology, Software, Writing – original draft. JZ: Investigation, Visualization, Writing – original draft. ZW: Resources, Writing – original draft. JM: Investigation, Resources, Writing – review & editing. SR: Investigation, Resources, Writing – review & editing. MX: Conceptualization, Funding acquisition, Software, Validation, Writing – review & editing. TH: Project administration, Supervision, Validation, Writing – review & editing.
